# Histone methyltransferase SMYD2: ubiquitous regulator of disease

**DOI:** 10.1186/s13148-019-0711-4

**Published:** 2019-08-01

**Authors:** Xin Yi, Xue-Jun Jiang, Ze-Min Fang

**Affiliations:** 1 Department of Cardiology, Renmin Hospital of Wuhan University, Wuhan, 430060 China; 2 Cardiovascular Research Institute, Wuhan University, Wuhan, 430060 China; 3 Hubei Key Laboratory of Cardiology, Wuhan, 430060 China; 4 Division of Cardiothoracic and Vascular Surgery, Tongji Hospital, Tongji Medical College, Huazhong University of Science and Technology, 1095 Jiefang Ave, Wuhan, 430030 China

**Keywords:** SMYD2, Methyltransferase, Nonhistone protein, Cardiovascular disease, Cancer

## Abstract

SET (Suppressor of variegation, Enhancer of Zeste, Trithorax) and MYND (Myeloid-Nervy-DEAF1) domain-containing protein 2 (SMYD2) is a protein methyltransferase that methylates histone H3 at lysine 4 (H3K4) or lysine 36 (H3K36) and diverse nonhistone proteins. SMYD2 activity is required for normal organismal development and the regulation of a series of pathophysiological processes. Since aberrant SMYD2 expression and its dysfunction are often closely related to multiple diseases, SMYD2 is a promising candidate for the treatment of these diseases, such as cardiovascular disease and cancer. Here, we present an overview of the complex biology of SMYD2 and its family members and their context-dependent nature. Then, we discuss the discovery, structure, inhibitors, roles, and molecular mechanisms of SMYD2 in distinct diseases, with a focus on cardiovascular disease and cancer.

Although histone methylation was discovered as early as 1964 [1, 2], it was not deeply investigated until the discoveries of the first histone methyltransferase (HMT) in 2000 and the first histone demethylase in 2004 [3, 4]. Histone methylation is written by HMTs and erased by histone demethylases [5]. HMTs are mainly divided into two categories according to methyltransferase activity on lysine or arginine residues, namely, protein lysine methyltransferases (PKMTs) and protein arginine methyltransferases (PRMTs) [6]. PKMTs consist of two classes: SET (Suppressor of variegation, Enhancer of Zeste, Trithorax) domain-containing PKMTs and non-SET-domain-containing PKMTs [7, 8], both of which are able to methylate lysine on its ε-amine group as mono (me1), di (me2), or tri (me3) methylation (Fig. 1) [6]. *S*-Adenosyl-l-methionine (AdoMet) is used as the primary methyl group donor to transfer one, two, or three methyl groups to lysine residues (Fig. 1) [9]. PRMTs are methyltransferases that mediate arginine-specific methylation. Arginine can be either monomethylated (MMA; Rme1), asymmetric dimethylarginine (ADMA; Rme2a), or symmetric dimethylarginine (SDMA; Rme2s) on one of the ω-amino groups [10]. In addition to histones, nonhistone proteins can also be methylated by PKMTs and PRMTs [11].

**Fig. 1:**
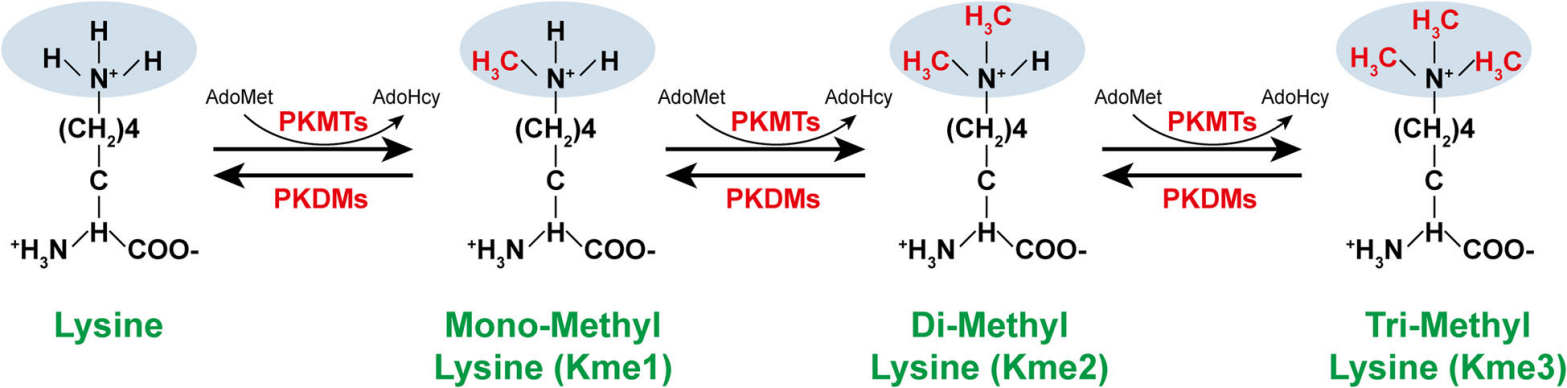
A schematic diagram of protein methylation on lysine residues. Protein lysine methyltransferases (PKMTs) catalyze monomethylation (Kme1), dimethylation (Kme2), and trimethylation (Kme3) of proteins on the ε-amine group of lysine by using *S*-adenosyl-l-methionine (AdoMet) as the primary methylgroup donor. This modification is reversible and can be erased by protein lysine demethylases (PKDMs)

There are five members of the SET and MYND (Myeloid-Nervy-DEAF1) domain-containing (SMYD) protein family, which is a special class of PKMTs that methylate both histones and nonhistone targets (Fig. 2) [8, 12, 13]. The SET and MYND domains are conserved in all five SMYD family members, and the SET domain is split into two segments (the S-sequence and a core SET domain) by the MYND domain [8, 14, 15]. The core SET domain is responsible for transferring methyl group to lysine residues on target proteins, while the S-sequence may participate in cofactor binding and protein-protein interactions [14]. The MYND domain which contains a zinc finger motif primarily plays a critical role in protein-protein interactions [16]. Another feature of this family is that all members have post-SET and SET-I domains, while the C-terminal domain (CTD) is found in only SMYD1-4 [8, 17]. The structure of the SMYD family has been detailed in a review published by Yang and colleagues [8]. Although SMYD family members have similar protein structure, their function and regulatory mechanisms in disease differ from one another. For example, Gottlieb et al. demonstrated that SMYD1 is a cardiac- and skeletal muscle-specific protein and mainly targets histone 3, lysine 4 (H3K4) methylation [18]. More importantly, SMYD1-deficient mice have defects in cardiomyocyte maturation and right ventricle formation [18]. Although SET and MYND domain-containing protein 2 (SMYD2) has the highest expression in the neonatal heart, it is dispensable for heart development in mice, in contrast to SMYD1 [19]. In addition, SMYD2 was demonstrated to be ubiquitously expressed in several tissues and to be an H3K36-specific methyltransferase that also targets H3K4 [17, 20]. Research advances in recent decades have highlighted SMYD family member involvement in development, cardiovascular disease, cancer, and other diseases by using various animal models, and several published reviews have summarized their functions and mechanisms [8, 12, 14, 17]. In the present review, we focus on only SMYD2, and systematically summarize research on SMYD2.

**Fig. 2:**
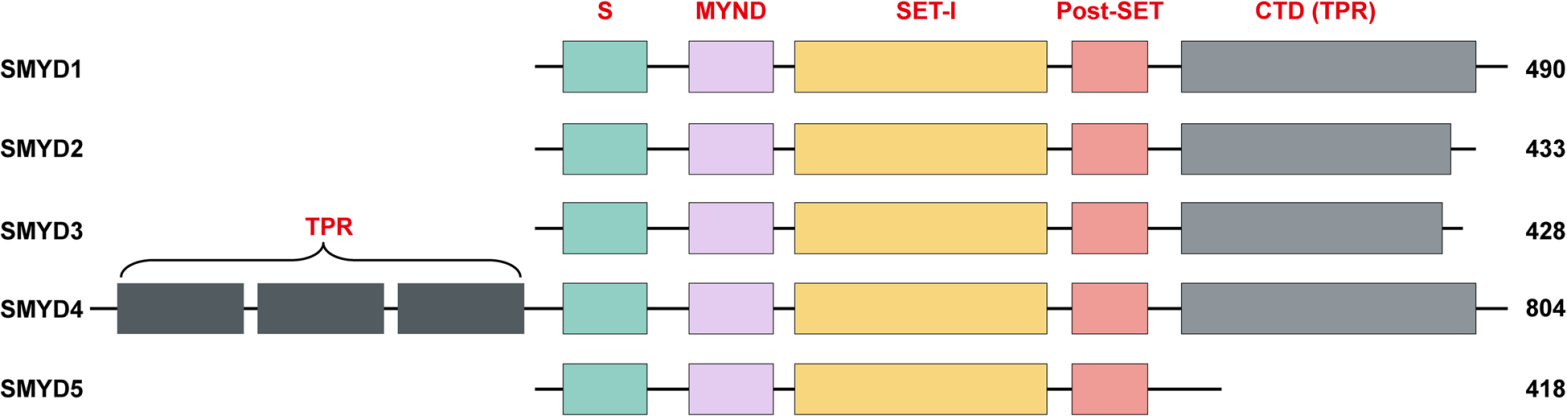
Schematic representation of SMYD family members. Linear representation of structural domains in SMYD1, SMYD2, SMYD3, SMYD4, and SMYD5. The domains are indicated as different colors, and the SET domain is the major catalytic domain. The numbers at the end represent the size of each respective SMYD protein in humans

## Discovery of SMYD2 and its structure

The histone methyltransferase Smyd2, located in the 1q32.3 region, was first identified by Brown and colleagues in 2006 [20]. Their study showed that Smyd2 mRNA levels are highest in the heart, brain, liver, kidney, thymus, and ovary by using northern blotting, and immunohistochemical staining demonstrated that SMYD2 localizes within both the nucleus and cytoplasm [20]. The crystal structure of full-length human SMYD2 was obtained by two independent research groups in 2011 [21, 22]. These results showed that the overall structure of SMYD2 is composed of five structurally distinct domains, including the S-sequence (1-46 aa), MYND domain (47-96 aa), insertion SET domain (97-243 aa), cysteine-rich post-SET domain (244-271 aa), and tetratrico-peptide repeat (TPR) domain (272-433 aa), which together form two large lobes that are separated by a deep groove [21]. The N-terminal region (1-271 aa) of SMYD2 contains a mixed structure of α-helices (α1-α6), β-strands (β1-β12), and long extended loops, and the C-terminal region (272-433 aa) is a twisted seven α-helical bundle (α8-α14) [22]. The S-sequence is important for optimal enzymatic activity of SMYD2, while the post-SET domain is essential for enzymatic activity as removing it completely abolishes enzymatic activity [23]. Metal analyses demonstrated that three zinc ions bind tightly to SMYD2 and are important for maintaining SMYD2 structural integrity and catalytic activity [23]. The methyltransferase activity of SMYD2 has been reported to be regulated by autoinhibition via intra- and interdomain bending of C-terminal domain (CTD) which stabilizes the autoinhibited conformation of SMYD2 and restricts substrates to access to the catalytic site via extensive engagement with the methyltransferase domain [24]. SMYD2 has a bilobal structure, and an open-closed motion of this structure may regulate substrate specificity [25]. When challenged with activation stress, AdoMet binding triggers increased elasticity of the CTD leading SMYD2 to adopt fully opened configurations to expose the substrate binding crevice [26]. Estrogen receptor alpha (ERα) binds SMYD2 in a U-shaped conformation, and the binding specificity determined mainly by residues in C-terminal to the target lysine and also by numerous intrapeptide contacts that ensures shape complementarity between the substrate and the active site of the enzyme [27]. Similarly, p53 binds to a deep pocket of the interface between catalytic SET and CTD with an unprecedented U-shaped conformation, and the tetratrico-peptide repeat motif of the CTD and EDEE motif between the loop of anti-parallel β7 and β8 sheets of the SET core may play an important role in determining p53 substrate binding specificity [28]. Modeling study of SMYD2 in complex with a p53 peptide indicates that monomethylation of p53-Lys372 mediated by SET7/9 might result in steric conflict of the methyl group with the surrounding residues of SMYD2 to inhibit SMYD2-mediated methylation of p53-Lys370 [22, 29]. In addition, AZ505, a substrate-competitive inhibitor of SMYD2, binds in the peptide binding groove of SMYD2 to confine other substrates binding [21]. Except for ERα and p53, SMYD2 methylates a spectrum of nonhistone proteins, and how the binding specificity of SMYD2 with its substrates is determined under certain conditions is a scientific question deserving in-depth investigation. Notably, the results of published literatures indicated that the broad specificity of SMYD2 is achieved by multiple molecular mechanisms such as distinct peptide binding modes and the intrinsic dynamics of peptide ligands [27].

## Nonhistone proteins methylated by SMYD2

SMYD2 was first identified as an H3K36-specific methyltransferase [20], and in a more in-depth study, SMYD2 was shown to not only methylate histone H3K4, but also methylate nonhistone proteins [23, 30]. For example, SMYD2 was reported to monomethylate p53 at K370 and pRb at K860 to inhibit the activities of these proteins [31, 32]. Recently, two research groups identified dozens of nonhistone substrates of SMYD2 via immunoprecipitation combined with mass spectrometry or bioinformatics, respectively [33, 34]. In Olsen’s study, they provided a global landscape of nonhistone protein methylation in esophageal squamous cell carcinoma (ESCC) cell with SMYD2 overexpression by using stable isotopic labeling with amino acids in cell culture (SILAC) coupled with immunoaffinity enrichment of monomethyl-lysine (Kme1) peptides and mass spectrometry [33]. In particular, they confirmed the identification of four novel SMYD2 substrates (BTF3, PDAP1, AHNAK, and AHNAK2) from 1861 Kme1 sites mapping to 1217 proteins. Ahmed et al. proposed a bioinformatics approach to identify novel cellular substrates of the lysine methyltransferase SMYD2. A total of 14 novel substrates were identified and six of which (MAPT, CCAR2, EEF2, NCOA3, STUB1, and UTP14A) were confirmed in cells by immunoprecipitation [34]. More importantly, several canonical nonhistone proteins, including p53 (K370me1) [31], HSP90 (K616me1) [35], PTEN (K313me1) [36], RB (K860me1 and K810me1) [32, 37], ERα (K266me1) [38], HSP90AB1 (K531me1 and K574me1) [39], STAT3 [40], p65 [40, 41], BMPR2 [42], MAPKAPK3 [43], ALK [44], and PARP1 [45], have been reported to be methylated by SMYD2 to affect cell proliferation, cellular differentiation, and cell survival. Thus, the substrates of SMYD2 are diverse, and methylated substrates mediated the function of SMYD2 on diseases, such as cardiovascular disease and cancer.

## Inhibitors of SMYD2

Analysis of the crystal structure of proteins is essential for drug research and development. Since the first crystal structure of SMYD2 was released in 2011, several inhibitors of SMYD2 have been developed by pharmaceutical companies [46]. AZ505, the first inhibitor of SMYD2 identified from a high-throughput chemical screen, shows greater selectivity toward SMYD2 than other KMTs (e.g., DOT1L and EZH2) with an inhibitory potency in the submicromolar range (IC_50_ = 0.12 μM) [21]. AZ505 binds in the peptide binding groove of SMYD2 to competitively inhibit the binding of substrates, such as p53 [21]. In 2015, Eli Lilly and Company developed the first cell-active, selective small molecule inhibitor of SMYD2, LLY-507, which has 100-fold selectivity for SMYD2 over 24 other methyltransferases [47]. The crystal structure of SMYD2 in complex with LLY-507 shows that this compound binds in the substrate peptide binding pocket, and it inhibits the ability of SMYD2 to methylate p53 with an IC_50_ < 15 nM [47]. More importantly, LLY-507 inhibits the proliferation of multiple tumor cell lines in a dose-dependent manner [47]. A-893 is another cell-active benzoxazinone inhibitor of SMYD2; this compound is a peptide-competitive inhibitor that exhibits high selectivity toward SMYD2 over a panel of 30 methyltransferases [48]. Furthermore, A-893 showed 80-fold more activity than AZ505 when tested by a biochemical assay [48]. BAY-598 is a potent and selective aminopyrazoline-based small molecule inhibitor of SMYD2 [49]. It is a substrate-competitive inhibitor of SMYD2 with greater than 100-fold selectivity toward SMYD2 in a panel of 32 methyltransferases. In vivo, BAY-598 can enhance the efficacy of doxorubicin on xenograft tumors [49]. In addition, AZ506 and EPZ033294 are inhibitors of SMYD2 that can inhibit the proliferation of cancer cell lines and p53 methylation [50, 51]. Although several types of SMYD2 inhibitors have been discovered, all of these existing inhibitors are based on the function of SMYD2 in p53 methylation and cancer. However, the function of SMYD2 extends beyond these two areas. Therefore, these SMYD2 inhibitors may have limitations and unpredictable side effects. Furthermore, most of these inhibitors have been tested in only in vitro systems, whether they have an effect on xenograft tumors, carcinoma in situ, or other diseases and underlying mechanisms require further investigation.

## SMYD2 in cardiovascular development and disease

**Fig. 3:**
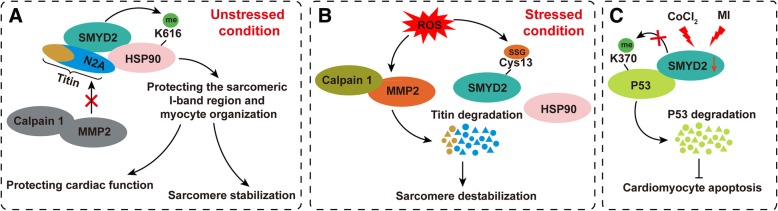
Mechanisms underlying the role of SMYD2 in cardiovascular disease. **a** Under non-stressed conditions, SMYD2 interacts with HSP90 and the N2A domain of titin to monomethylate HSP90 at lysine 616 (HSP90K616me1), and this complex can prevent the degradation of titin by MMP2 and Calpain 1. On the other hand, this complex protects the sarcomeric I-band region and myocyte organization to maintain cardiac function and sarcomere stabilization. **b** Upon stimulation with reactive oxidative species (ROS), SMYD2 is glutathionylated at Cys13, which leads to the dissociation of Hsp90 and titin. Then, titin is degraded by activated MMP2 to affect sarcomere destabilization. **c** Upon stimulation with cobalt chloride (CoCl_2_) or myocardial infarction (MI), the expression level of SMYD2 is decreased, which results in a reduction in p53 methylation (p53K370me1), increased p53 stability, and the induction of cardiomyocyte apoptosis.

Given the high level of SMYD2 expression in the heart [20], its roles in heart development and cardiac disease were seriously considered after it was discovered. To investigate the role of SMYD2 in heart development, Diehl et al. generated a cardiac-specific SMYD2 knockout mouse model (SMYD2-cKO) that developed a normal heart [19]. Morphologic analysis demonstrated that SMYD2-cKO mice have a comparable heart to body weight ratios as their control littermates at 6 months of age; moreover, no obvious differences in necrosis, cardiomyocyte organization, or cardiac fibrosis, as detected by hematoxylin and eosin (HE) staining and Masson’s trichrome staining, were observed between SMYD2-cKO and control mice. In addition, the mice with or without SMYD2 deficiency had similar cardiac function, which was assessed by MRI-analysis [19]. Thus, these results indicate that SMYD2 is dispensable for mouse heart development, while it was reported that SMYD2 knockdown results in developmental delay and aberrant tail formation in zebrafish [52]. In contrast, the highly related paralogue SMYD1 plays important roles in heart development and cardiomyogenesis, as evidenced by the right heart ventricle formation defect in SMYD1 null mice [12]. SMYD3 also affects heart development in zebrafish [53]. SMYD2 does not affect heart development in mice, but Voelkel et al. reported that knockdown of SMYD2 in zebrafish leads to heart defect and cardiac dysfunction [35]. Their results demonstrated that SMYD2 deficiency zebrafish showed malformation of both the atrium and ventricle and also presented pericardial edema, elongation and thinning of the hearts, inflow tract edema, and reduced heart rate and cardiac function. Mechanistically, in the cytoplasm of cardiomyocytes, SMYD2 monomethylates Hsp90 at lysine 616 and interacts with the sarcomeric I-band region at the titin N2A-domain via its N-terminal and extreme C-terminal regions to influence cardiac contraction [35]. Similarly, in muscle, SMYD2 methylates Hsp90 to maintain the stability of titin and muscle function (Fig. 3a) [54]. This discrepancy in heart development between mice and zebrafish may be due to the following reasons: (1) differences in genetic background between species, (2) other SMYD family members compensating for a lack of SMYD2 function in mice, and (3) Nkx2-5-cre which was used to generate cardiac-specific knockout SMYD2 mice in Diehl’s study is expressed in cardiac progenitor in early embryo (only label myocardial and endothelial cells within the left and right ventricles) [19, 55], which does not excluded the possibility that SMYD2 affects heart development via regulating other cardiac cells, such as cardiac progenitor in anterior heart field (Mef2c-AHF-Cre) or cardiomyocyte in late embryo (cTNT-Cre) [55]. It was reported that EZH2, another histone methyltransferase, plays different roles in heart when knockout it in different cardiac cells by distinct cardiac-specific Cre mice. For example, the results of EZH2^myh6-cre^ and EZH2^cTNT-Cre^ knockout mice demonstrated that EZH2 is dispensable for heart repair [56], but EZH2^Nkx2-5-cre^ knockout mice displayed later disruption of cardiomyocyte gene expression and heart development [56], and EZH2^mef2c-AHF-Cre^ knockout mice showed postnatal myocardial pathology [57].

A recently study demonstrated that SMYD2 is selectively glutathionylated at cysteine 13 in response to reactive oxygen species, and SMYD2 glutathionylation or oxidation decreases the viability of H9c2 cells and reduces myofibril integrity [58]. After oxidation or glutathionylation at cysteine 13 of SMYD2, Hsp90 and titin dissociate from SMYD2, and titin is degraded by activated MMP2 to affect sarcomere stability (Fig. 3b) [58]. Furthermore, SMYD2 was reported to act as an endogenous antagonist of p53-dependent cardiomyocyte apoptosis [59]. In cobalt chloride-induced neonatal rat ventricular cardiomyocytes, SMYD2 expression levels are significantly decreased, and knockdown of SMYD2 promotes cobalt chloride-induced cardiomyocyte apoptosis by affecting p53 methylation and stability. Moreover, cardiac-specific deletion of SMYD2 in mice promotes apoptotic cell death upon myocardial infarction (Fig. 3c) [59]. All the abovementioned studies indicate that SMYD2 is important for cardiomyocyte survival and cardiac function. However, whether SMYD2 regulates the pathophysiology of the heart in response to other stresses, such as pressure overload, ischemia-reperfusion injury, and diabetic cardiomyopathy, requires further investigation.

Interestingly, the role of SMYD2 in abdominal aortic aneurysm (AAA) was highlighted by a meta-analysis of genome-wide association studies (GWAS) [60]. Four new AAA risk loci, 1q32.3 (SMYD2), 21q22.2 (ERG), 13q12.11 (LINC00540), and 20q13.12 (near PCIF1/MMP9/ZNF335) were identified via a meta-analysis that included six GWAS data sets and a validation study totaling 10,204 cases and 107,766 controls [60]. More importantly, Toghill et al. assessed global DNA methylation in peripheral blood mononuclear cell DNA from 92 AAA cases and 93 controls; their data showed that four CpGs (NC_000001.11: 214280412, 214280441, 214280507, and 214280600) were hypomethylated in the SMYD2 promoter upstream of the transcriptional start site in AAA patients compared to controls [61]. Importantly, the mRNA and protein expression levels of SMYD2 were significantly downregulated in larger AAA patients and SMYD2 promoter methylation has a direct linear relationship with SMYD2 expression [61]. As SMYD2 gene expression was not correlated with aortic diameter of individuals with AAA greater than 55 mm, these results indicated that SMYD2 may be associated with the development but not progression of AAA [61]. Furthermore, SMYD2 has been reported as a novel negative regulator of macrophage activation and M1 polarization, and these macrophages secrete a large amount of proinflammatory cytokines. Overexpression of SMYD2 in macrophages accelerates TGF-β production and suppresses IL-6 secretion by inhibiting Th-17 cell differentiation while promoting regulatory T cell differentiation [62]. Given that increased inflammatory responses and inflammatory cell infiltration are key hallmarks of AAA pathobiology, decreased SMYD2 levels in AAA patients may facilitate disease progression via reactivation of macrophages and inflammation. Hsp90 may also mediate the role of SMYD2 in AAA formation because Hsp90 is monomethylated by SMYD2, and the inhibition of Hsp90 could reduce AAA formation in murine models [63]. However, the exact role and molecular mechanisms of SMYD2 in AAA or even aortic dissection remain unclear. A myeloid cell- or vascular smooth muscle cell (VSMC)-specific SMYD2 knockout mouse model is needed to ascertain the function of SMYD2 in AAA/aortic dissection and other cardiovascular diseases.

## SMYD2 in cancer

**Fig. 4:**
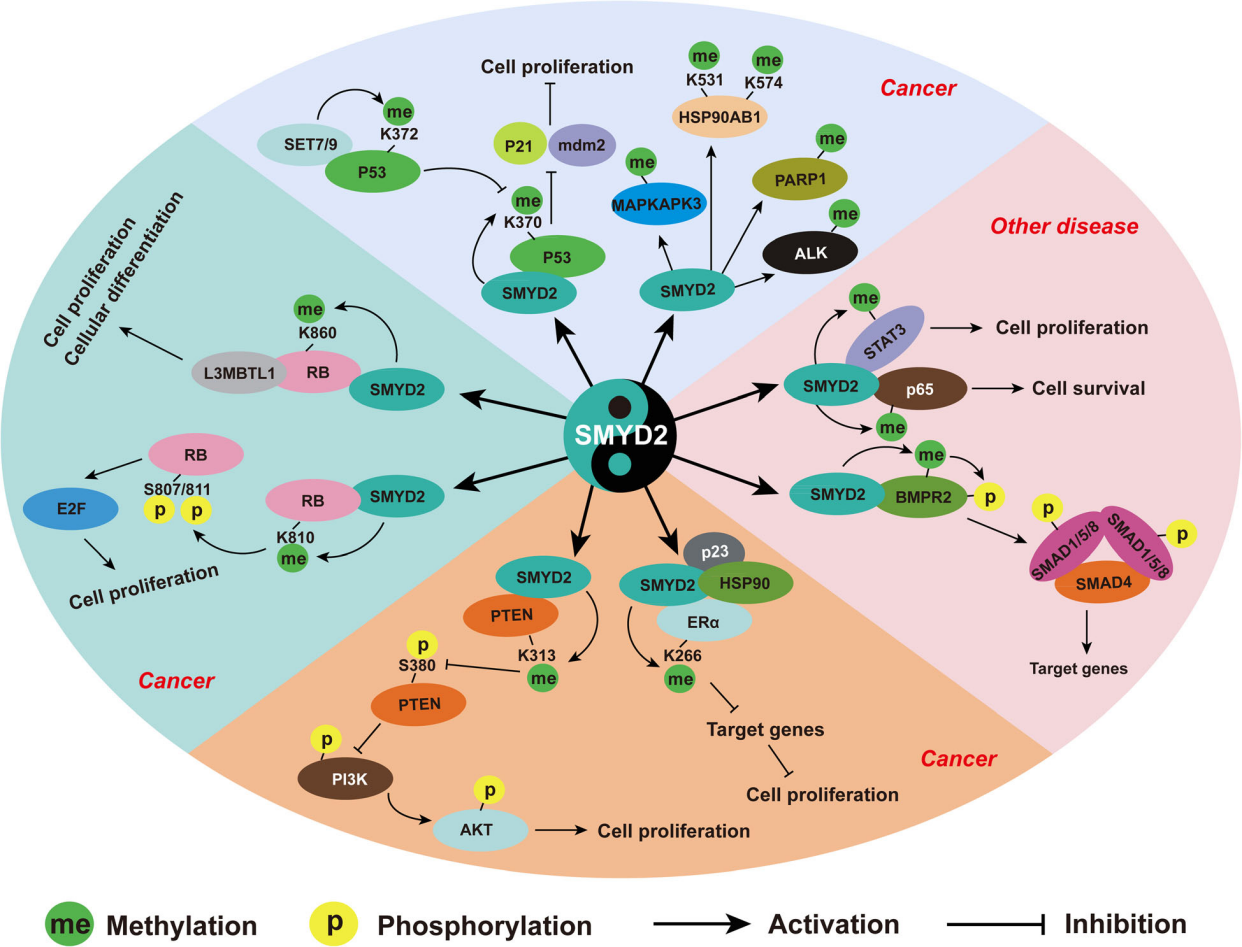
The nonhistone substrates of SMYD2 involved in cancer and other disease. Several nonhistone proteins are methylated by SMYD2 during cancer formation, including p53, RB, PTEN, ERα, PARP1, ALK, MAPKAPK3, and HSP90AB1. SMYD2 methylates and activates p65 and STAT3 to regulate autosomal dominant polycystic kidney disease. BMPR2 methylated by SMYD2 to activate SMAD1/5 signaling. Methylation on substrates always cross-talks with other posttranslational modifications, especially phosphorylation, to affect signaling pathways and target genes related to cancer and other disease

In addition to cardiovascular diseases, the role of SMYD2 in cancer has received broader attention. p53, the first identified nonhistone protein substrate of SMYD2, is the most famous tumor suppressor [31]. SMYD2 monomethylates p53 at Lys370 (p53K370me1) to inhibit p53 activity and thus suppress p21 and Mdm2 expression to facilitate cancer cell proliferation. However, Set9-mediated p53 methylation at Lys372 (p53K372me1) could inhibit SMYD2-mediated methylation of Lys370 by blocking the interaction between p53 and SMYD2 [31]. The results of high-resolution crystal structures showed that p53 binds to a deep pocket of the interface between catalytic SET and the CTD domain of SMYD2 with an unprecedented U-shaped conformation [24, 28]. A growing number of studies have revealed the important role of SMYD2 in several types of cancer, including breast cancer, leukemia, esophageal squamous cell carcinoma, and gastric cancer (Fig. 4) [41, 64–66].

Breast carcinoma (BC), one of the most common types of malignancy, is the second leading cause of cancer associated mortality among women [67]. Therefore, early screening and diagnosis are very important. A research group recently investigated the expression patterns and prognostic value of SMYD family members in human BC patients by using data released in a public database [68]. Their results demonstrated that compared with normal individuals, BC patients had significantly increased mRNA levels of SMYD2/3/5 and reduced mRNA levels of SMYD1/4. Among these family members, SMYD2 mRNA expression level was associated with the relapse-free survival of BC patients with metastatic relapse, indicating that SMYD2 is a potential biomarker and prognostic indicator for the diagnosis of BC [68]. In breast cancer cell lines and tissues, Li et al. demonstrated that SMYD2 expressed at higher levels; furthermore, knockdown of SMYD2 in triple-negative breast cancer (TNBC) cell lines or inhibition of SMYD2 activity by AZ505 significantly reduced tumor growth in vivo. Mechanistically, SMYD2 promotes TNBC cell proliferation and survival via methylation and activation of STAT3 and the p65 subunit of NF-κB [41]. Estrogen signaling plays important roles in cell growth and differentiation, and its dysregulation leads to many human diseases, including a variety of human cancers (e.g., breast and ovarian cancer) [69]. Estrogen receptor alpha (ERα), a ligand-activated transcription factor, recruits a number of coregulators to estrogen response elements to modulate gene activation or repression in response to estrogen stimulation. More importantly, ERα is highly expressed in breast and ovarian tissues [70]. Thus, revealing the regulatory mechanisms of ERα expression and activity is critical for understanding human diseases. In MCF7 breast cancer cells, Zhang et al. demonstrated that SMYD2 directly methylates ERα at Lysine 266 (K266) to prevent ERα target gene activation [38]. The crystal structure revealed that ERα binds SMYD2 in a U-shaped conformation and the binding specificity is determined mainly by residues in the CTD domain of SMYD2 [27]. In addition, SMYD2 interacts with the molecular chaperones HSP90/p23 to increase the methylation of Lysine 266 in ERα [71]. In addition to ERα, phosphatase and tensin homolog (PTEN) is also a substrate of SMYD2 in breast cancer cells. SMYD2 methylates PTEN at Lysine 313 in vitro and in vivo to negatively regulate PTEN tumor suppressor activity, resulting in activation of the phosphatidylinositol 3-kinase (PI3K)-AKT pathway. In SMYD2 knockdown breast cancer cells, AKT phosphorylation levels are attenuated, while PTEN phosphorylation at Serine 380 is increased. These results indicate that SMYD2-mediated methylation of PTEN at Lysine 313 diminishes the phosphorylation of PTEN at Serine 380, which subsequently results in AKT activation to promote breast cancer cell growth [36]. Ovarian carcinoma is another common malignant tumor among women. Kukita et al. found that SMYD2 expression levels are significantly increased in high-grade serous ovarian carcinoma (HGSOC) clinical tissues, and knockdown of SMYD2 or inhibition of SMYD2 with LLY-507 accelerates apoptotic cell death. Moreover, LLY-507 showed an additive effect with the poly (ADP-ribose) polymerase (PARP) inhibitor olaparib in colony-formation assays, indicating that LLY-507 can be used alone or in combination with olaparib for the treatment of HGSOC patients [72]. PARP1 is considered an important target for the development of anticancer therapy, and its inhibitor, olaparib, was developed for BRCA1/2 mutated HGSOC [73, 74]. Piao et al. reported that SMYD2 monomethylates and activates PARP1 to enhance poly (ADP-ribose) formation under conditions of oxidative stress [45]. Thus, the synergistic effect of LLY-507 and olaparib against HGSOC may be associated with the role of SMYD2 in PARP1 methylation. The abovementioned studies indicate that SMYD2 is overexpressed in BC and HGSOC patients, and SMYD2 may function as a potential biomarker for diagnosing these cancers. More importantly, the inhibition of SMYD2 could be a very efficient therapeutic strategy for BC and HGSOC; however, more specific inhibitors should be developed, and multicenter clinical trials should be performed to validate this hypothesis.

The retinoblastoma (RB), a tumor suppressor and central cell cycle regulator, is methylated by SMYD2 at Lysine 860, which creates a direct binding site for the transcriptional repressor L3MBTL1; together, these proteins regulate cell cycle progression, cellular differentiation, and in the response to DNA damage [32]. Interestingly, in addition to Lysine 860, Lysine 810 of RB is also methylated by SMYD2, which enhances Serine 807/811 phosphorylation of RB, and mediates the role of SMYD2 in bladder cancer cell growth [37]. Moreover, methylated RB accelerates E2F transcriptional activity and promotes cell cycle progression. Most importantly, the expression level of SMYD2 is significantly increased in human bladder carcinoma compared with nonneoplastic bladder tissues, which indicates that inhibitors of SMYD2 may have a therapeutic effect on bladder carcinoma [37]. Higher SMYD2 mRNA expression level is observed in renal cell tumors than in normal renal tissues, and SMYD2 mRNA levels discriminated renal cell tumors from normal renal tissue with 100% specificity and 82.1% sensitivity and distinguished chromophobe subtype renal cell carcinoma (chRCC) from oncocytoma, with 73.3% specificity and 71.0% sensitivity [75].

In esophageal squamous cell carcinoma (ESCC), overexpression of SMYD2 protein is observed in most primary tumor samples (76.5%) and in some ESCC cell lines (25.6%). ESCC patients with SMYD2 overexpression have a lower overall survival rate than those with low SMYD2 expression [65]. Knockdown of SMYD2 significantly inhibits ESCC cell growth (KYSE150 and KYSE790) [65]. More importantly, Olsen et al. used stable isotopic labeling with amino acids in cell culture (SILAC) coupled with immunoaffinity enrichment of monomethyl-lysine (Kme1) peptides and mass spectrometry to identify substrates of SMYD2 in ESCC cells. They identified 1861 Kme1 sites in SMYD2-overexpressing ESCC cells, 35 of which were potently downregulated by both SMYD2 knockdown and SMYD2 inhibition by LLY-507 [33]. Although the authors did not further investigate the downstream pathways mediating the function of SMYD2 in ESCC, this large-scale proteomic study of lysine monomethylation regulated by SMYD2 provides useful data for future studies. Approximately 60–70% of patients with human papillomavirus (HPV)-unrelated head and neck squamous cell carcinoma (HNSCC), another type of squamous cell carcinoma, have SMYD2 overexpression, and these patients have a worse overall survival rate than those with low SMYD2 expression [76].

Acute lymphoblastic leukemia (ALL) is the most common malignancy in children. Chimeric antigen receptor T cell (CAR-T) therapy is gradually gaining popularity in treating certain types of leukemia, but chemoradiotherapy is currently preferred as no large-scale clinical trials of CAR-T therapy have been completed now. High SMYD2 expression is correlated with a poor prognosis in ALL patients, and patients with low SMYD2 expression levels are more sensitive to chemotherapy [64]. SMYD2 is directly activated by the transcription factor MYC, and ablation of SMYD2 impedes the development of leukemia promoted by the fusion oncogene MLL-AF9 [77]. In addition, SMYD2 knockdown confers relative resistance of human acute myeloid leukemia cells to multiple classes of DNA damaging agents, which is associated with the compensatory upregulation of SET7/9 [78]. Similarly, in chronic lymphocytic leukemia (CLL) patients, SMYD2 and SMYD3 are overexpressed, accompanied by a high white blood cell count and complex karyotype [79].

In addition, SMYD2 is also overexpressed in gastric cancer [66], pancreatic ductal adenocarcinoma (PDAC) [43], papillary thyroid carcinoma [80], hepatocellular carcinoma [81], and colon cancer [82]. Furthermore, overexpression of SMYD2 protein positively correlates with larger tumor size, more aggressive lymphatic invasion, deeper tumor invasion, and higher TNM stage, as well as worse overall survival rate [80, 81]. Multiple nonhistone proteins are methylated by SMYD2 to achieve these effects on tumors, for example, MAPKAPK3 in PDAC and ALK in non-small-cell lung cancer (NSCLC) [43, 44]. SMYD2 also methylates HSP90AB1 at lysines 531 and 574 to accelerate the proliferation of cancer cells [39].

Although many studies have reported the role of SMYD2 in tumors, most of these studies have analyzed the SMYD2 expression pattern in tumors using data released in public databases. SMYD2 overexpression has been observed in all the types of tumors investigated, and knockdown or inhibition of SMYD2 suppresses tumor cell growth by methylating different substrates in different cancers. However, a recently published study offered a different conclusion; this study used CRISPR/Cas9 to genetically ablate SMYD2, and the results indicated that SMYD2 activity is dispensable for autonomous cancer cell proliferation [51]. More experiments should be performed to verify this conclusion, but this study prompts us to seriously consider the specificity of inhibitors and off-target effects when performing genetic manipulations.

## SMYD2 in other diseases

The role of SMYD2 in cardiovascular disease and cancer has been extensively investigated, but its function in other diseases remains largely unknown. Li et al. found that SMYD2 is upregulated in renal epithelial cells, kidney tissues from Pkd1 mutant mice, and autosomal dominant polycystic kidney disease (ADPKD) patients [40]. SMYD2 deficiency largely reverses renal cyst growth in Pkd1 mutant mice by suppressing the methylation and activation of STAT3 and p65 to inhibit cystic renal epithelial cell proliferation and survival (Fig. 4) [40]. RNAi-based screening of human lysine methyltransferases that regulate HIV-1 latency demonstrated that knockdown of SMYD2 reactivates latent HIV-1 in both T cell lines and primary CD4^+^ T cells via the SMYD2-H4K20me1-L3MBTL1 axis [83]. Autophagy is important for maintaining the homeostasis of organisms, and excessive autophagy is associated with disease. SMYD2 deficiency enhance autophagic cell death induced by BIX01294, an inhibitor of EHMT2 [84]. Inflammation plays a critical role in multiple diseases and biological processes. SMYD2 functions as a negative regulator of macrophage activation to inhibit the production of proinflammatory cytokines [62]. In addition, two environmental toxins, bisphenol A and phthalates, affect macrophage activation by regulating SMYD2-mediated H3K36 modification [85]. Recently, SMYD2 was reported to regulate the bone morphogenic protein (BMP) signaling pathway [42]. SMYD2 specifically methylates the kinase domain of BMP type II receptor 2 (BMPR2) and then activates the BMP-induced phosphorylation of SMAD1/5 and subsequent nuclear localization and interaction with SMAD4 (Fig. 4) [42].

## Conclusions and perspectives

The recent surge of data on SMYD2 structure and function highlights that SMYD2 is a critical regulator of cardiovascular disease and cancer. Given that SMYD2 is overexpressed in multiple cancers, it may be a broad-spectrum therapeutic target for cancer treatment, and a valuable biomarker for diagnosis and prognosis. Interestingly, most studies have demonstrated that SMYD2 methylates nonhistone proteins, not histones, to achieve its function, which is consistent with the fact that SMYD2 primarily localizes to the cytoplasm. More importantly, the methylation of nonhistone proteins always cross-talks with other posttranslational modifications, especially phosphorylation. In addition, SMYD2 plays diverse roles in different cells, organs, and cancers by regulating distinct substrates. Thus, side effects may be unavoidable if SMYD2 inhibitors are administered orally or through intravenous injection. Therefore, the delivery of inhibitors by using drug carriers with different affinities for target organs or cells will be a direction of future research. Additionally, a conditional SMYD2 knockout or overexpression mouse model will be useful for investigating the role of SMYD2 in development or disease. There is an urgent need to clarify the function of SMYD2 under distinct conditions and to ascertain how the specificity of SMYD2 binding to a specific substrate is determined. These investigations, together with the development of more potent and specific SMYD2 inhibitors, hold promise for the treatment of prevalent tumor types and cardiovascular diseases.

## Data Availability

Not applicable.

## Abbreviations

AAA: Abdominal aortic aneurysm
AdoMet: *S*-Adenosyl-l-methionine
ADPKD: Autosomal dominant polycystic kidney disease
ALL: Acute lymphoblastic leukemia
BC: Breast carcinoma
BMPR2: BMP type II receptor 2
CAR-T: Chimeric antigen receptor T cell
chRCC: Chromophobe subtype renal cell carcinoma
CLL: Chronic lymphocytic leukemia
CTD: C-terminal domain
ERα: Estrogen receptor alpha
ESCC: Esophageal squamous cell carcinoma
GWAS: Genome-wide association studies
H3K4: Histone 3, lysine 4
HE: Hematoxylin and eosin
HGSOC: High-grade serous ovarian carcinoma
HMT: Histone methyltransferase
HNSCC: Head and neck squamous cell carcinoma
HPV: Human papillomavirus
MYND: Myeloid-Nervy-DEAF1
NSCLC: Non-small-cell lung cancer
PARP: Poly (ADP-ribose) polymerase
PDAC: Pancreatic ductal adenocarcinoma
PI3K: Phosphatidylinositol 3-kinase
PKMTs: Protein lysine methyltransferases
PRMTs: Protein arginine methyltransferases
PTEN: Phosphatase and tensin homolog
RB: Retinoblastoma
SET: Suppressor of variegation, Enhancer of Zeste, Trithorax
SMYD2: SET and MYND domain-containing protein 2
TNBC: Triple-negative breast cancer
TPR: Tetratrico-peptide repeat
VSMC: Vascular smooth muscle cell
